# Recombination, Diversity and Allele Sharing of Infectivity Proteins Between *Bartonella* Species from Rodents

**DOI:** 10.1007/s00248-012-0033-y

**Published:** 2012-03-15

**Authors:** Anna Paziewska, Edward Siński, Philip D. Harris

**Affiliations:** 1 National Centre for Biosystematics, Natural History Museum, University of Oslo, PO Box 1172, Blindern, Oslo, Norway; 2 Department of Parasitology, Institute of Zoology, Faculty of Biology, University of Warsaw, Miecznikowa 1, 02-096 Warsaw, Poland

## Abstract

The alpha-Proteobacterium *Bartonella* is a common parasite of voles and mice, giving rise to short-lived (4 weeks to 2 months) infections. Here, we report high sequence diversity in genes of the VirB/VirD type IV secretion system (T4SS), amongst *Bartonella* from natural rodent populations in NE Poland. The VirB5 protein is predicted to consist of three conserved alpha helices separated by loops of variable length which include numerous indels. The C-terminal domain includes repeat stretches of KEK residues, reflecting underlying homopolymeric stretches of adenine residues. A total of 16 variants of VirB5, associated with host identity, but not bacterial taxon, were identified from 22 *Bartonella* isolates. One was clearly a recombinant from two others, another included an insertion of two KEK repeats. The *virB5* gene appears to evolve via both mutation and recombination, as well as slippage mediated insertion/deletion events. The recombinational units are thought to be relatively short, as there was no evidence of linkage disequilibrium between *virB5* and the *bepA* locus only 5.5 kb distant. The diversity of *virB5* is assumed to be related to immunological role of this protein in *Bartonella* infections; diversity of *virB5* may assist persistence of *Bartonella* in the rodent population, despite the relatively short (3–4 weeks) duration of individual infections. It is clear from the distribution of *virB5* and *bepA* alleles that recombination within and between clades is widespread, and frequently crosses the boundaries of conventionally recognised *Bartonella* species.

**Electronic supplementary material:**

The online version of this article (doi:10.1007/s00248-012-0033-y) contains supplementary material, which is available to authorized users.

## Introduction

The alpha-Proteobacterium *Bartonella* includes such significant human pathogens as *Bartonella quintana*, *Bartonella henselae* and *Bartonella bacilliformis*. However the greatest diversity is found amongst rodent-infecting forms; of more than 1,400 citrate synthase (*gltA*) sequences within GenBank, over 530 are from rodents. Rodents are infected at high prevalence (sometimes exceeding 60 %, see Ref. [1]), by co-existing *Bartonella* species [1–6] which undergo genetic exchange both within and between taxa [7–11], and these bacteria form a significant component of the parasite community infecting rodents, although their impact on rodents appears to be minimal [6, 12]. Most significantly, these rodent-infecting forms may represent an important reservoir for emergent strains of *Bartonella* which infect humans [13–17]. The host specificity of *Bartonella* is unclear. At one level, species appear to lack specificity [10], and opportunistic human infections with, e.g. *Bartonella vinsonii* or *Bartonella grahamii* suggest that many *Bartonella* taxa may have a wide host range. On the other hand, while species may lack host specificity, individual clades within species show close fidelity to particular genera of host rodents [11], suggesting that research on host specificity has focussed at an inappropriate taxonomic level.

*Bartonella* has a three-phase life cycle [18]. An initial proliferative phase infecting endothelial cells persists for several days, after which bacteria escape to invade erythrocytes, within which they remain until either the death of the cell or uptake in the blood meal of an insect. In laboratory rodents, lesions in liver and kidneys have also been observed [19], although their relevance to the epidemiology of natural *Bartonella* infections is unknown. The third phase of the life cycle is within the gut of an insect vector. Invasion of both endothelial and red blood cells depends upon type IV secretion systems (T4SS), encoded within pathogenicity islands in the *Bartonella* genome [20, 21]. The T4SS of *Bartonella* is similar to the type A T4SS of other gram-negative bacteria [22, 23], but levels of homology with other bacteria are low [24]. T4SS gene clusters have been duplicated and lost during genomic rearrangements in *Bartonella*, but typically two or three systems are present [20]; *trw*, responsible for red blood cell infection [24] and *virB*/*virD* and *vbh*, involved in endothelial cell infection [20, 25]. The *trw* system in *Bartonella* is unique in that several genes within the cluster are duplicated [24, 26]. Sequencing of the *B. grahamii* genome [21] revealed an astonishing diversity of pathogenicity genes, and, including those encoded by a plasmid, the sequenced isolate could access a total of 69 T4SS genes, compared to none in *B. bacilliformis* [20, 21].

**Figure 1:**
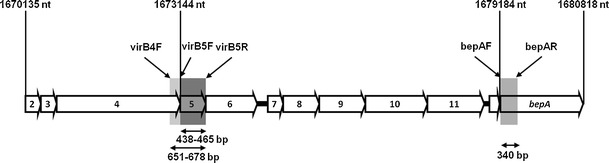
Schematic drawing of *virB* gene complex showing both *virB5* and *bepA* genes, based on data available for *B. grahamii* (isolate as4up) genome (accession number: NC012846, see [21]); *virB* (2–11) and *bepA* genes marked, with primers used to amplify *virB5* gene and *bep*A gene fragment, and the size of amplicons. Positions in genome (*nt*) based on isolate NC012846

Despite the evidence from whole genome sequencing, and experimental knowledge of T4SS function [20, 27], little is known of the diversity of T4SS genes in natural *Bartonella* infections. Here, we present evidence of the molecular diversity and mode of evolution of two T4SS genes, *virB5* (encoding a putative adhesin involved in host cell invasion) and *bepA*, located 5.5 kb from *virB5* (see Fig. 1) and encoding a crucial inoculated effector protein which prevents host cell apoptosis [28], in *Bartonella* isolates circulating in rodent populations in an old field and forest system in North East Poland.

## Materials and Methods

The field study site represents a complex of forest and abandoned agricultural land in NE Poland, the focus of several previous studies of rodent-pathogen epidemiology [6, 11, 29–36]. This work follows [37], concentrating on a longitudinal field study of rodents in an old field habitat, where the voles *Microtus oeconomus* and *Microtus arvalis* and the mouse *Apodemus flavicollis* were collected, and a forest, where an area of 2 ha was sampled monthly for *A. flavicollis* and *Myodes glareolus* [6, 11, 36] from June 2007 until May 2009, with short breaks during winter (December–March). Detailed trapping, marking and collection protocols have been presented elsewhere [6, 11, 38]. After collection of blood and ectoparasites, the rodents were marked with an ear tag (World Precision Instruments Inc., USA) and released close to their original capture point. Animal handling and sampling methodologies conformed to permission granted by the Polish Local Ethical Committee (737/2007). Blood (50–100 μl) was taken by caudal vein bleeding into 1 mM EDTA and stored at 4°C prior to DNA extraction using a proprietary method (AxyPrep Miniprep Blood, AxyGen, USA). A 316 bp fragment of the *gltA* gene was amplified using primers Bh.CS782p and Bh.Cs1137n [39]. Further data were collected from cultures of *Bartonella* isolated from rodents euthanised with an ether overdose, and then bled by cardiac puncture. The blood was stored in 1 mM EDTA at 4°C until use, then plated on Columbia blood agar and incubated at 37°C in a 5 % CO_2_ atmosphere for up to 10 days [40]. Each isolate was subcultured three times before single colonies were selected. Cells were harvested and DNA isolated by boiling in sterile water for 10 min, and centrifuging at 12,000 rpm for a further 15 min [41]. The supernatant was stored frozen and used with the following primers to amplify genes of the T4SS-*bepA* (bepAF: 5′-TCATCATTAATTTTATCCGAACACCAC-3′, bepAR: 5′-TGAGATAAATTCTTCGCGCGTTAA-3′) and *virb5*. The first pair of primers (virB5F: 5′-TGGAATGAATGATGAGATCGCC-3′ and virB5R: 5′-TAAAGTCGGACATCAGAWTTCYCAAG-3′) was used on all isolates but failed to consistently amplify *virB5*. The forward primer (virB4F: 5′-TACAGTCAGAATTAAGCAGAG-3′) was therefore designed to complement the 3′ end of the *virB4* gene (immediately 5′ to *virB5*; see Fig. 1) and used with the *virB5* reverse primer (virB5R), to successfully amplify *virB5* from a further 12 isolates. In the PCR reaction, after first denaturation in 94°C for 300 s, genes were amplified with 45 cycles of 94°C for 45 s, 51°C (using primer pair bepAF/bepAR) or 52°C (virB5F/virB5R and virB4F/virB5R) for 45 s, 72°C for 60s, and followed by a single 7-min extension step at 72°C. Sequencing used amplification primers on both strands of purified amplicons (AxyPrep™ Clean, AxyGen, USA or ExoSAP-IT®, Affymetrics, USA) with ABI technology (Genomed, Poland or ABI-lab, University of Oslo, Norway). New sequences obtained in this study were deposited in GenBank under accession numbers: HM450047–HM450063 and JN797608-JN797610 (*virB5*), and HM450064–HM450072 (*bepA*); see Electronic Supplementary Material.

Sequence outputs were checked visually for ambiguous peaks, which, where appropriate, were re-sequenced. Because of indels within the *virB5* gene, it proved difficult to achieve an unambiguous alignment, and therefore three approaches to alignment were adopted. The first was to use standard and Expresso alignment algorithms within the T-Coffee package [42]. The second was to use the Prank algorithm via the WebPrank server (www.ebi.ac.uk/). The final approach was by homology modelling using the *Escherichia coli* TraC protein as a template [43, 44] via the SWISS-MODEL package (swissmodel.expasy.org [45–47]). The modelled protein structures were then used as a guide for alignment of the DNA sequences. Maximum Likelihood and Bayesian phylogenetic analysis, including analyses of selection on codon positions and of rates of evolution of different lineages, was carried out using PhyML and PAML [48, 49], after choosing the best DNA evolution model (jModelTest [50]). Nested clade analysis was performed as described [51–53], using log-linear models (SPSS) to test statistical hypotheses concerning the segregation of *virB5* and *bepA* variation with clade identity or host identity. Analysis of recombination was carried out using the RDP-3 software package [54].

## Results

A total of 37 variant *gltA* sequences were recovered from 154 *Bartonella* strains isolated over 2 years from four rodent species [11]. Most could be referred either to *B. grahamii* (five variants) or to *Bartonella taylorii* (26 variants), but one isolate of *Bartonella birtlesii* and three of *Bartonella doshiae* were also identified. Two variants had recombinant *gltA* genes [11]; one of these (Mo1) was *B. doshiae*-like at other housekeeping gene loci. The characterisation of *virB5* and *bepA* genes was attempted only for the 42 *Bartonella* isolates cultured on blood agar. *VirB5* was amplified from 30 isolates, *bepA* from 17; both sequences were obtained from 12 isolates.

Out of 30 isolates for which *virB5* was amplified, 20 were *B. taylorii* clades, six were *B*. *grahamii*, three were *B. doshiae* (including one recombinant) and one *B. birtlesii* [11], according to *gltA* gene identity. In all, the inferred VirB5 protein product was between 145- and 173-amino acids (aa) in length; amplicons from *B. taylorii* and *B. birtlesii* isolates were clearly shorter than those from non-recombined *B. doshiae* and *B. grahamii* isolates (inferred length 145–152 and 148 aa vs. 159 and 173 aa, respectively). Because of apparent similarities of sequence, the *Bartonella elizabethae* sequence from GenBank (AF195504) was included in the alignment. The *B. grahamii* isolate from Uppsala (GenBank accession number NC012846) proved identical to *B. grahamii* isolates collected in the present work (Mg4, Af4). Homology modelling of *Bartonella* VirB5 molecules suggested that this protein is made up of three helices, a short helix at the carboxy terminus following the signal peptide, followed by two long helices separated by a loop. The most reliable structures generated were for *B. elizabethae* and *B. taylorii* (Table 1). The model for *B. elizabethae* (QMEAN4 = 0.7, Z-score = −1.39, see [55]), featured a long (24 aa) loop between helices 2 and 3. The poorest fit model was for *B. grahamii*, which could not be modelled using the automatic algorithm. However, a manual alignment, based on the alignment of *B. elizabethae* to TraC, and the Prank alignment of *B. grahamii* to *B. elizabethae*, allowed the generation of the same protein structure as in the case of the other *Bartonella* species (Table 1). All VirB variants sequenced during the present work could be modelled with the same structure, with variable length loops due to indels between helices 2 and 3, and at the end of helix 3 (Table 1). Helices 1, 2 and 3 represented the most highly conserved parts of the translated molecule.

**Table 1:** Properties of VirB5 proteins as deduced from SWISS-MODEL homology modelling, using *Escherichia coli* Trac1 as template

Strain	QMEAN4 score	Z-score	Length (aa) (position in the protein (aa))			Length (aa) of the loop between helixes 2 and 3	Indel(s) position(s) in the protein (aa)	Protein length (aa)
			Helix 1	Helix 2	Helix 3			
*Bartonella elizabethae* (AF195504)	0.7	−1.4	11 (29–39)	25 (45–69)	31 (101–131)	30	72–89	177
							92–93	
							97–103	
							151–153	
							157–160	
							171–172	
*Bartonella grahamii* (NC012846)	0.4	−4.1	11 (29–39)	25 (45–69)	31 (98–128)	28	72–85	173
							88–89	
							93–99	
							147–149	
							153–156	
							167–168	
*Bartonella doshiae* (Ma16)	0.5	−2.6	11 (29–39)	25 (45–69)	35 (81–115)	11	72–75	159
							81–87	
							149–155	
*Bartonella birtlesii* (Af5)	0.6	−2.0	11 (29–39)	25 (45–69)	31 (81–111)	11	72–78	148
*Bartonella taylorii* (Af1)	0.7	−1.4	11 (29–39)	25 (45–69)	29 (80–108)	10	72–75	145
*B. taylorii* (Ma12)	0.6	−1.8	11 (29–39)	25 (45–69)	31 (81–111)	11	72–78^a^	148
*B. taylorii* (Mo2)	0.6	−1.8	11 (29–39)	25 (45–69)	32 (80–111)	10	72–78^a^	154
							140–145	
*B. taylorii* (Af6)	0.6	−1.5	11 (29–39)	25 (45–69)	31 (81–111)	11	72–78^a^	148
*B. taylorii* (Ma13)	0.5	−2.5	11 (29–39)	25 (45–69)	36 (80–115)	10	72–78	152
							84–87	

Indel positions marked following the alignment obtained using prank algorithm

^a^Identical amino acid indels

### Phylogenetic Reconstruction of *virB5*

**Figure 2:**
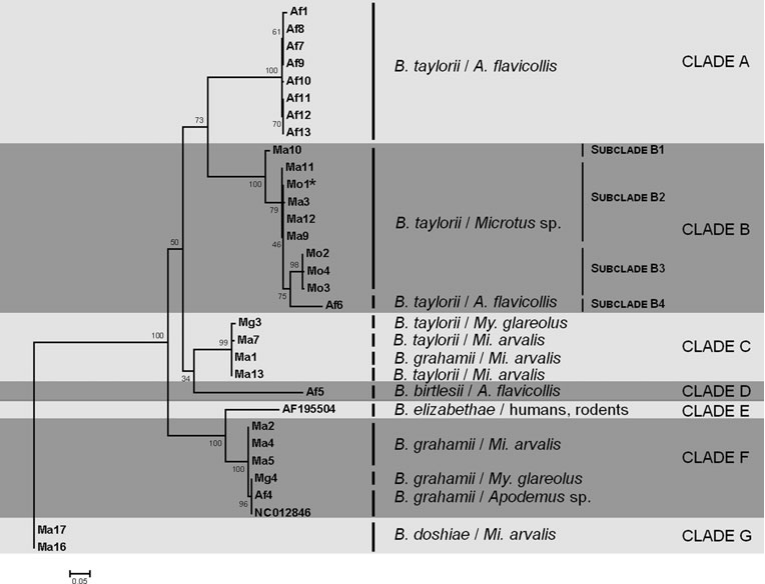
Phylogeny of obtained sequences of *virB5* gene (465 bp), generated using PhyML with a GTR substiution model and the Prank amino acid alignment. Isolate identified as recombinant on basis of housekeeping genes (see Ref. [11]) marked with an *asterisk*

Phylogenetic reconstructions using alignments generated using different algorithms did not differ in the shape of resultant trees generated using maximum likelihood (PhyML), although the alignment did affect the maximum likelihood estimates of the trees, with Prank with a GTR model (estimated using jModelTest) giving the best estimate (ML = −2,769). This alignment was therefore used in subsequent analyses. A phylogenetic reconstruction using this alignment divided the 16 basic variants into six clades (Fig. 2), corresponding to *B. doshiae*, *B. grahamii*, *B. birtlesi*, two clades of *B. taylorii* and one mixed clade of *B. taylorii* and *B. grahamii*. When included, *B. elizabethae* made a 7th clade, linked as sister group to the *B. grahamii* clade. The *virB5* clades were structured according to host species infected. Thus, clade B in Fig. 2 infected mainly voles from the field system, although it also included one isolate from *A. flavicollis* from forest. Clade C consisted of four isolates from voles, three from *M*. *arvalis* and one from *M. glareolus*. Clade F was collected from three *M. arvalis* from the fallows, and from one representative of each of the forest rodents, *M. glareolus* and *A. flavicollis*. Two clades (A, eight isolates, and D, one isolate, see Fig. 2) were found only in *A. flavicollis*. This association of *virB5* alleles with host identity was significant (*χ* ^2^ = 33.8, *df* = 15, *P* = 0.004).

Differences between clades were much greater than those within clades, and were due to mutation, recombination and loss/gain of indels. Within clade A, isolates differed by up to two nucleotides; only Af1 (13–14 bases difference from the rest of clade A isolates) could not have been a result of clonal expansion. Clades C and F varied by, respectively, up to five and three bases while both clade G isolates were identical. Clade B was most diverse, with four subclades (Fig. 2). Subclade B1 consisted of only one recombinant isolate from *M. arvalis* (see below). Subclade B2, from both *Microtus* species (Ma3, Ma9, Ma11, Ma12 and Mo1), varied by up to three bases. Subclade B3 isolates from *Mi. oeconomus* differed by up to two bases, and were sister group to B2, with a characteristic insertion of 6-amino acids (aa 148–153, LKKLEK) in the carboxy terminus of the protein generated by an imperfect repetition of two sections of DNA (TTAAAA and GAAAAA). Subclade B3 alleles were not recently derived from subclade B2, as there were 16 other mutational changes between these alleles. The fourth subclade B4, consisted only of the isolate Af6. By other phylogenetic approaches (Maximum Parsimony) this isolate is basal to the entire tree, and it is very different in sequence to the other clade B isolates. Its position within this clade is therefore uncertain.

The isolate Ma10 from subclade B1 (Fig. 2), proved to be recombinant between a clade A allele and an allele from subclade B2 (infecting *Apodemus* and *Microtus*, respectively), with a breakpoint between positions 73 and 77 of the DNA sequence. Statistical support was high (*P* = 7.4 × 10^−11^, RDP; 2.9 × 10^−30^, GENECONV; 2.3 × 10^−5^, BootScan; 3.2 × 10^−5^, Chimera; 3.5 × 10^−5^, MaxChi; 3.2 × 10^−29^, SiScan; 3.3 × 10^−16^, 3 Seq). Visual inspection showed 17 base changes, shared between clade A and the Ma10 isolate but differing from subclade B2, up to position 73 and 73 changes from position 77 to the end of the gene, which were shared between Ma10 and subclade B2 but not with clade A. There were also indels of 18 bases between positions 223 and 240 and of 12 bases between positions 391 and 402, shared between Ma10 and subclade B2 but absent from clade A. Only a single base change (G436T) appeared to have occurred in Ma10 which was not shared with either clade A or subclade B2.

Differences between clades were due to both mutational change and to indels. Each clade had a characteristic pattern of indels, which could account for up to 100 bases difference between the shortest and the longest sequences (Table 1). Nevertheless, the clades also differed substantially due to mutational change, with more than 130 base changes (c. 30 % of the gene) between the most diverse isolates. Mutational change and indels were correlated, and phylogenies based on alignments in which indels were omitted had the same topology as those for which they were included.

### BepA

**Figure 3:**
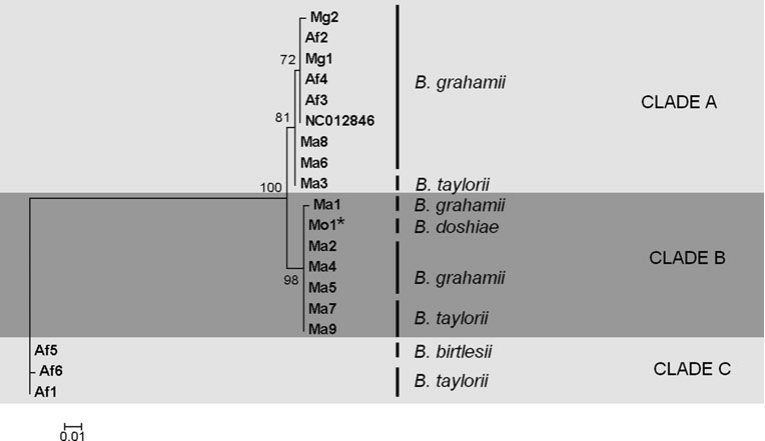
Phylogeny of obtained sequences of *bepA* gene fragment (340 bp), generated using PhyML with a GTR substition model and the Prank amino acid alignment. Recombinant isolate (see Ref. [11]) marked with an *asterisk*

Only a fragment of the *bepA* gene was amplified, between amino acids 26 and 138 of the complete protein, encompassing the Fic-1 domain as identified by Schulein et al. [28]. In the present work, *bepA* amplified successfully from 17 *Bartonella* isolates, of which five could be classified as *B. taylorii*, ten as *B. grahamii* and one as *B. birtlesii* [11]. The final isolate which amplified was Mo1, the *B. doshiae* isolate with a recombinant *gltA*. Compared with *virB5*, there was relatively little variation in *bepA* at either nucleotide or amino acid level, and the fragments proved easy to align and to belong to three clades of sequence variants (Fig. 3). No relationship was found between host identity or bacterial species identity. Clade A was amplified from eight isolates (predominantly *B. grahamii* with a single *B. taylorii* isolate) collected from *M. arvalis* (three isolates), *M. glareolus* (two isolates) and *A. flavicollis* (three isolates). The *bepA* from the fully sequenced Swedish *B. grahamii* isolate (NC012846), also belonged to this clade. Only two single nucleotide polymorphisms occurred within this group, one silent, the other substituting Q60P (numbering based on NC012846 [21]). Clade B was made up of six isolates from *M. arvalis* (two *B. taylorii* and four *B. grahamii*) and one from *Mi. oeconomus* (the *B. doshiae* with recombinant *gltA*). Six isolates within this clade had identical nucleotide sequences but one of the *M. arvalis* isolates (Ma1) differed by a single nucleotide causing an inferred amino acid change (L54M, numbering according to NC012846 [21]). Clades A and B were five nucleotides different, with a characteristic E121K substitution. The third *bepA* clade was however completely different, with 49 nucleotide changes relative to clades A and B, resulting in 19- and 18-amino acid substitutions, respectively. It was found in three isolates, two referrable to *B. taylorii* and the third to *B. birtlesii*, all from *A. flavicollis*. Within this group, there was a single silent nucleotide substitution (A309G in Af6; DNA numbering based on NC012846 [21]).

**Figure 4:**
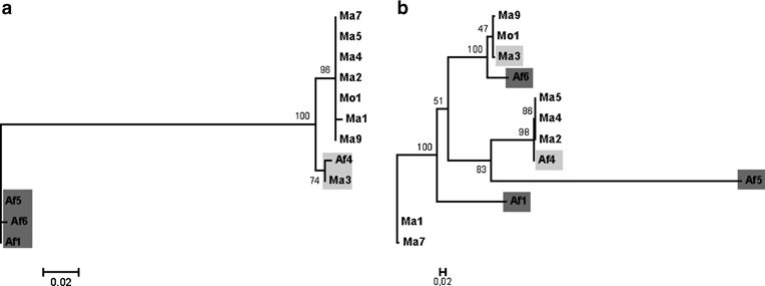
Phylogenetic trees of isolates, for which both **a** *bepA* and **b** *virB* gene sequences were obtained, generated using PhyML with a GTR substition model and the Prank amino acid alignment. Isolates of particular clades of *bepA* (**a**) marked with the same colors on *virB*5 tree (**b**)

For the 12 isolates where both *virB5* and *bepA* were successfully amplified, there was no consistent pattern of linkage between alleles (Fig. 4). For example, Af1 (*B. taylorii*) and Af5 (*B. birtlesii*) shared a *bepA* allele but exhibited the most disparate *virB5* alleles, suggesting that recombination between these loci is common, despite their close proximity (no more than 5.5 kb apart [21]). Alleles of both *bepA* and *virB5* were distributed between all *Bartonella* spp. as established using housekeeping genes [11]. There was however no consistent phylogenetic pattern in the distribution of *virB5* alleles (see Table 2). For example, identical *virB5* alleles amplified from isolates Ma1 and Ma7, which were entirely different at all sequenced housekeeping gene loci except *groEl*. Conversely, isolate Mo1, identical with both *B. doshiae* isolates (Ma16 and Ma17) according to housekeeping genes except for a recombinant *gltA* [11], had an entirely distinct *virB5* allele.

**Table 2:**
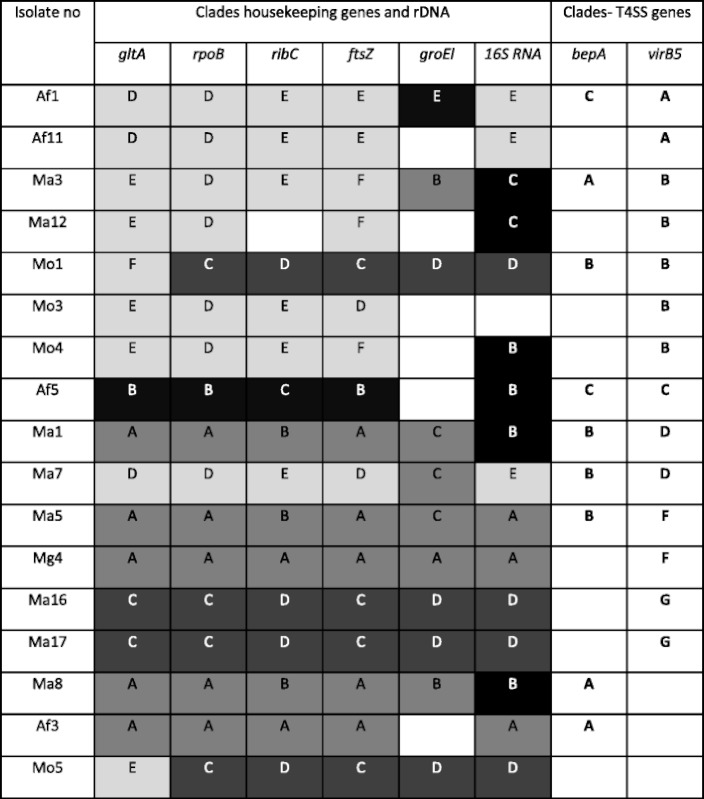
Allele patterns (chosen housekeeping genes, rDNA and T4SS gene fragments) of *Bartonella* isolates from different *virB5* clades

Bacteria species based on housekeeping genes and 16S rDNA; shade colour—from the brightest: *Bartonella taylorii*, *Bartonella grahamii*, *Bartonella doshiae* and *Bartonella birtlesii*

### Distribution and Recombinant Pathways of *bepA* and *virB5* Within Rodent Populations

**Figure 5:**
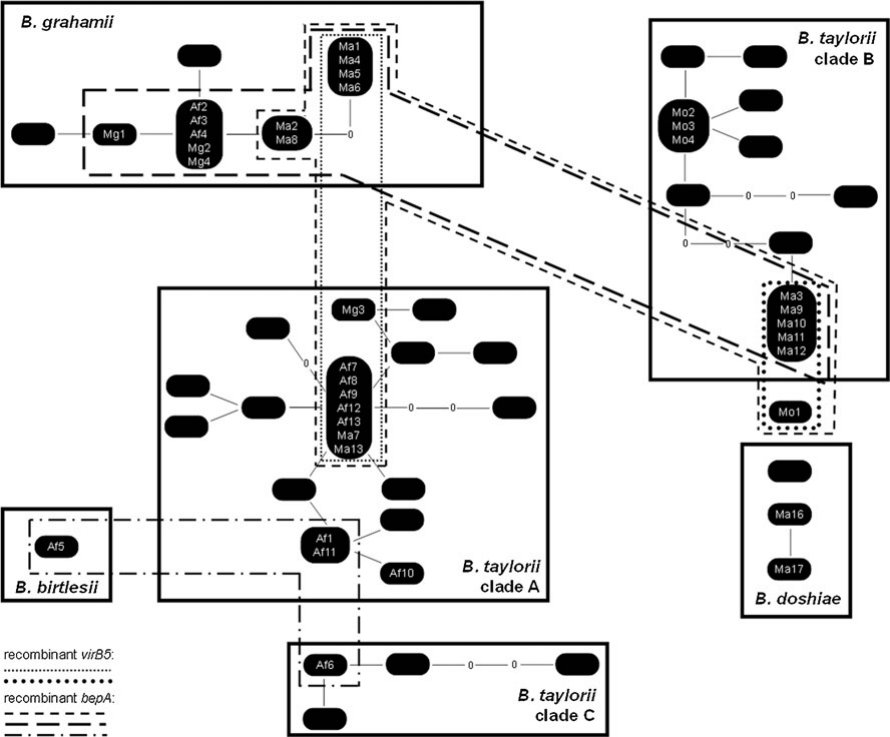
Cladogram of the *Bartonella* isolates collected from rodents at the study site (as described in Ref. [11]), and relevant haplotypes from Welc-Falęciak et al. [37], based on 292 bp fragment of *gltA*, showing recombinant events of *virB5* and *bepA*. *Black boxes* indicate variants of *gltA* that have been found in the study, ‘missing’ variants (i.e. predicted but not collected) are indicated by 0. Isolates for which sequencing of *virB5* and/or *bepA* were successfully marked on (*Af A. flavicollis*, *Mg M. glareolus*, *Ma M. arvalis*, *Mo M. oeconomus*). Clades sharing *virB5* and *bepA* variants between ‘species’ and/or main clades of *B. taylorii* are marked. Main clades (*B. grahamii*, *B. taylorii* clades A, B and C, and *B. doshiae*) are based on 95 % probability that all isolates within them have arisen through mutation (calculated in TCS [56])

Analysis of *gltA* demonstrated a complex of *Bartonella* species and clades which recombine with each other to varying degrees ([11]; Fig. 5). *VirB5* variants (clade F on Fig. 2) from clades of *B. grahamii* diverged from each other by no more than three nucleotides, and were congruent with clonal expansion inferred from *gltA*. *BepA* variants from *B. grahamii* were also consistent with this hypothesis. *VirB5* clade A variants were characteristic of *B. taylorii gltA* clade A (see Fig. 2), and were also sufficiently similar to indicate clonal expansion. However, a second clade of *virB* variants (Clade C in Fig. 2) also occurred within *B. taylorii gltA* clade A (Ma7 and Ma13); these two forms could not have resulted from clonal diversification alone. One *virB* clade C variant (Ma1) was also recovered from *B. grahamii*, indicating recombination of the whole *virB5* gene between these species. *VirB5* clade B had the most interesting distribution within the *Bartonella* network. Most isolates (Ma3, Ma9, Ma11 and Ma12) came from a single *gltA* variant within *B. taylorii* clade B (see Fig. 5), with recombinant links to *B. doshiae* (see [11]); Mo1 was recombinant between *B. taylorii* and *B. doshiae* [11]. The somewhat different *virB5* subclade B3 from Mo2, Mo3 and Mo4 also circulated within *B. taylorii gltA* clade B (Fig. 5). The presence of identical *bepA* alleles suggested a recombinant pathway between *B. taylorii gltA* clade B, *B. grahamii* and the recombinant *B.taylorii/B. doshiae* isolate Mo1. Finally sharing of the basal *bepA* allele C between *B. birtlesii* and *B. taylorii* clade A suggests that these isolates may also recombine.

## Discussion

The *Bartonella* genome is extensively reshaped by recombination, both at a genomic scale [10, 20, 21] and more locally at the level of individual genes [11]. Indeed, Nysted et al. [9] focussed on the *virB5* analogue at the *trw* locus to demonstrate duplication and recombination within a single clade of *B. grahamii*, identifying potentially recombinant stretches derived interspecifically (*B. henselae* sequences within the *B. grahamii* and *Bartonella tribocorum* genes) or from an unknown donor. Here, we present the first account of *virB5* evolution in a natural *Bartonella* population and show the relative importance of recombination and mutation in the evolution of this gene. Part of the pressure for recombination is thought to come from selection for optimal combinations of invasion genes, particularly through microevolution of adhesins such as BadA [10, 57] and of the T4SS cell invasion systems. We demonstrate that the sequence of *virB5* (identified as an adhesin by [44]) is linked to host identity, that its structure is shaped by recombination, and that it is exchanged between *Bartonella* clades and species which are only distantly related. Although this represents the first demonstration of such extensive intragenic rearrangements within *Bartonella*, in fact this process is well known in the related alpha-Proteobacterium *Helicobacter pylori*. Within a relatively small number of isolates of *virB5*, we have identified one clear example of recombination between two clades, another where the subclades have been derived by acquisition or loss of an indel of 7-amino acids based on a partial DNA repeat, a third clade derived by loss of 3-amino acids at the C terminus, and other examples where recombination or loss/gain of indels can be suspected in the origin of the genes. At the same time, there is extensive mutational drift between *virB5* clades showing different patterns of indels, although there is little mutational change within them. It should be stressed that we present here the minimum diversity of *virB5* likely to exist within the *Bartonella* community analysed, as a further 12 isolates failed to sequence, suggesting the existence of further variants.

Although VirB5 in other alpha-Proteobacteria is a three-helix, 220-amino acid acidic protein [44] of which the *E. coli* TraC protein is a prototype [43], the inferred *Bartonella* VirB5 protein is much shorter (145–177 aa), consisting of one short and two long helices. The second long helix, and the C terminal domain, both contain numerous charged residues and are the most variable parts of the molecule, including numerous indels. Although Backert et al. [44] make the case for VirB5 being involved in docking with integrins on the host cell surface, this seems not to be the case for the *Bartonella* VirB5, as the fibronectin recognition domain of the three-helix VirB5 molecules is absent [44]. However, the large number of charged residues in the C terminal domain of the *Bartonella* VirB5 protein does suggest a role in intra-molecular interactions. This could be related to the binding of the VirB5 molecule into the pilus formed by the T4SS [18]; in *B. henselae* VirB5 binds strongly to VirB3 [58], and in *Agrobacterium* this protein is an integral part of the pilus structure [59]. Additionally, this protein is a strong and specific immunogen in *Bartonella* infections, being characterised as the 17 kDa antigen in clinical studies [60]. Naturally immune sera had highly specific recognition patterns, suggesting exposure of native VirB5 to the host immune system [60], and making it likely that VirB5 of *Bartonella* is exposed on the outer surface of the pilus, where it has a species-specific conformation. In its alternation of runs of charged lysine and glutamate residues, VirB5 in *Bartonella* shows interesting parallels with the immunologically relevant glutamate-rich merozoite surface proteins of *Plasmodium* (e.g. [61, 62]). It is also similar in this respect to the VirB10/CagY protein of *H. pylori*, which includes runs of lysine and glutamate doublets and may be implicated in immunological evasion [63].

Evolution of *virB5* in *B. taylorii* has clearly taken place through homologous recombination, and at least one (subclade B1) of the seven *virB5* clades identified in this work is unambigiously derived by recombination from two others (B2 and A). The double duplication of 3-amino acids in subclade B3 is presumably due to misalignment of the repetitive (CTGAGAAA) motif between partner chromosomes during a recombinant event. The longest VirB5 molecules, those of *B. grahamii* and *B. elizabethae* have also evolved by duplication of short segments, with an insertion of 9-amino acids built up partly from a (GAAAAA) repeat. A second, imperfect (GCTTCAA) repeat (*n* = 3) gives a further insertion of 12-amino acids. Imperfect copying of partial repeat regions within *virB5* appears to have been an important mechanism for the microevolution of this gene. The *virB5* gene has numerous poly-A tracts, up to a maximum of A_8_, at which slippage during replication can occur [64]. This provides considerable plasticity to gene structure, and the presence of large numbers of glutamate and lysine residues may be related both to an antigenic function of VirB5, and to the importance of homopolymeric tracts at which slippage can occur.

Apart from small-scale duplications and evidence of recombination within the gene, there was also clear evidence of recombinant exchange of the whole *virB5* gene between *B. taylorii* and *B. grahamii*. This corresponded exactly with exchange of the housekeeping gene *groEl* between the same two clades of *B. taylorii* and *B. grahamii* [11], although it is not clear how much more of the genome was swapped between these isolates. Sharing of *virB5* variants showed little or no correlation with the patterns of *gltA* alleles present in the *Bartonella* isolates, further suggesting widespread recombination between isolates. However, the distribution of *virB5* variants correlated much more closely with host identity than did *gltA*. Recombination on a large scale in the *Bartonella* genome has been obvious since the comparisons of Frank et al. [65] and Saenz et al. [20], showing different genome sizes and gene arrangements across the genus, and a role for phage mediated recombination [21] was suggested following the observation of phage transcription in *B. bacilliformis* [66] and *B*. *grahamii* [21], packaging genomic fragments of c. 14 kb. The work of Nystedt et al. [9] also suggested recombination in a *virB5* homologue (*trw*), in this case inferred from a phylogenetic context. There was little evidence in the present work for linkage disequilibrium between *virB5* and the *bepA* gene, and phylogenies based on the two genes are quite different, although these genes are only 5.5 kb apart [21]. This further suggests that recombination involves relatively small stretches of DNA, certainly no larger than the 14-kb fragments packaged by the *Bartonella* gene transfer agent (GTA) [21]. Barbian and Minnick [66] indicate that not all *Bartonella* species possess this GTA, as evidenced by the presence or absence of a 14-kb extra-chromosomal band in whole genomic DNA preparations. *B. grahamii* does possess this GTA (confirmed by Berglund et al. [21]), but *Bartonella claridgeiae*, *B*. *vinsonii* and *B. elizabethae* do not; *B. taylorii*, *B. birtlesii* and *B. doshiae* have not been studied, and therefore we do not know whether all of the clades of *Bartonella* included in the present work had the GTA and were capable of recombination via this route.

The present work demonstrates a close parallel between *Bartonella* isolates in rodents and the epidemiological situation in *Helicobacter*, with a difference in the temporal scale of persistence of variants. In *H. pylori*, novel antigenic variants [63, 67, 68] appear within the same host, infected with *H*. *pylori* for perhaps 30 years. These variants allow the *H. pylori* infection to escape from host immunological surveillance [67], and take advantage of transient infection with co-colonising strains to undertake recombination [69]. In the context of *Bartonella* in rodents, individual infections are transient, lasting only 4–11 weeks [6, 70, 71], in rodents which live a maximum of 13–15 months [6]. However, genetic exchange and recombination within *virB5*, a molecule known to be antigenically relevant, probably allows numerous *Bartonella* isolates to circulate within the wider rodent population; temporal persistence in this case is replaced by spatial persistence. Clearly, the role of the arthropod vectors in generating and maintaining allelic diversity through recombination in *Bartonella* is important, and requires further elucidation. This is particularly important given the reputation that rodent *Bartonella* isolates are acquiring as emerging human pathogens [72].

## Electronic Supplementary Material

Below is the link to the electronic supplementary material.

ESM 1 (DOC 51 kb)
