# Supplementary material for: Recombination, Diversity and Allele Sharing of Infectivity Proteins Between Bartonella Species from Rodents
Source: Microb Ecol. 2012 Mar 15;64(2):525–36. doi: 10.1007/s00248-012-0033-y (PMC3391547; doi:10.1007/s00248-012-0033-y)
Supplement: Supplementary file 1 — (DOC 51 kb) [file 248_2012_33_MOESM1_ESM.doc]

Recombination, diversity and allele sharing of infectivity proteins between *Bartonella* species from rodents.

*Microbial Ecology*

Anna Paziewska, Edward Siński & Philip D. Harris

Corresponding author: Anna Paziewska, National Centre for Biosystematics, Natural History Museum, University of Oslo, PO Box 1172, Blindern, Oslo, Norway, e-mail: a.k.paziewska@nhm.uio.no

Online Resource 1. Accession numbers of *virB* gene and *bepA* and *gltA* gene fragments of isolates used in the study.

| Number of isolate | Accession number | | |
| --- | --- | --- | --- |
|  | *virB5* | *bepA* | *gltA* |
| Af1 | HM450047 | HM450072 | identical to AY435108 |
| Af2 | - | HM450068 | identical to Z70016 |
| Af3 | - | HM450068 | identical to Z70016 |
| Af4 | identical to NC012846 | HM450068 | identical to Z70016 |
| Af5 | JN797609 | HM450072 | identical to AF204272 |
| Af6 | HM450051 | HM450071 | GU338962 |
| Af7 | HM450048 | - | GU228956 |
| Af8 | HM450048 | - | GU338956 |
| Af9 | HM450048 | - | GU338956 |
| Af10 | HM450049 | - | GU338952 |
| Af11 | HM450050 | - | identical to AY435108 |
| Af12 | HM450050 | - | GU338952 |
| Af13 | HM450050 | - | GU338952 |
| Mg1 | - | HM450069 | GU338942 |
| Mg2 | - | HM450064 | identical to Z70016 |
| Mg3 | HM450061 | - | GU338964 |
| Mg4 | identical to NC012846 | - | identical to Z70016 |
| Ma1 | HM450062 | HM450070 | GU338946 |
| Ma2 | JN797610 | HM450065 | GU338945 |
| Ma3 | HM450056 | HM450067 | GU338968 |
| Ma4 | JN797610 | HM450065 | GU338946 |
| Ma5 | JN797610 | HM450065 | GU338946 |
| Ma6 | - | HM450067 | GU338946 |
| Ma7 | HM450063 | HM450065 | GU338957 |
| Ma8 | - | HM450067 | GU3389945 |
| Ma9 | HM450060 | HM450065 | GU338968 |
| Ma10 | HM450052 | - | GU338968 |
| Ma11 | HM450059 | - | GU338968 |
| Ma12 | HM450058 | - | GU563801 |
| Ma13 | HM450062 | - | GU338957 |
| Ma16 | JN797608 | - | GU338973 |
| Ma17 | JN797608 | - | GU338974 |
| Mo1 | HM450057 | HM450066 | GU338976 |
| Mo2 | HM450053 | - | identical to FJ946856 |
| Mo3 | HM450054 | - | identical to FJ946856 |
| Mo4 | HM450055 | - | identical to FJ946856 |
